# Evaluation of the pediatric-centered integrated care AOK Junior: protocol for a mixed-method study

**DOI:** 10.1186/s12913-020-05088-7

**Published:** 2020-03-16

**Authors:** Sebastian Liersch, Kathrin Krüger, Carina Oedingen, Andrea Spreenberg, Torben Bergemann, Christian Krauth

**Affiliations:** 1 AOK Nordost – Die Gesundheitskasse, Wilhelmstr. 1, 10963 Berlin, Germany; 2 Hannover Medical School, Institute for Epidemiology, Social Medicine and Health Systems Research, Carl-Neuberg-Str. 1, 30625 Hanover, Germany

**Keywords:** Health economic evaluation, Prevention, Children

## Abstract

**Background:**

The “AOK-Junior” care program of the AOK Nordost (a German statutory health insurance) completes the primary care for children and adolescents (C&A) in the federal states of Berlin, Brandenburg and Mecklenburg-Vorpommern in Germany. The focus of this program is on prevention and early detection of illness on C&A. Furthermore, the aim is to maintain the health of C&A and to prevent, detect and treat illness on time. Elements of the program are not only the integrated care of C&A, but also, for example, weight reduction and additional medical checkups U10, U11 and J2. The evaluation of the complex intervention should provide information about the effectiveness of early disease detection and costs-effectiveness as well as of other parameters like satisfaction.

**Methods:**

The evaluation is performed on the levels of structural-, process- and results-quality. The cost effectiveness is also assessed by means of a health economic evaluation. In addition to the collection of qualitative and quantitative primary data from participating and non-participating C&A and paediatricians, routine data from a statutory health insurance are used in the evaluation. Furthermore, a cross-sectional design is used to evaluate the structure and process quality. The effectiveness is evaluated in longitudinal section design on the basis of the secondary data. The quantitative surveys include net *n* = 1096 C&A and *n* = 340 pediatricians. For the focus groups, a sample of 72 to 96 parents as well as pediatricians will be sought by using the method of theoretical sampling.

**Discussion:**

Around 560 pediatricians and 63,000 C&A currently participate in the AOK Nordost care program. The project provides information to what extent secondary preventive measures can lead to the early detection of diseases and on the associated cost-effectiveness. Furthermore, potentials and barriers of the program implementation are identified. The results of the evaluation study are expected not only to contribute to the further development of the care program, but also to derive recommendations for action.

**Trial registration:**

German Clinical Trials Register (DRKS), DRKS-ID: DRKS00015280. Prospectively registered on 18 March 2019*.*

## Background

Medical care in childhood and adolescence is key to health development, the formation of health-promoting or –endangering behaviour, and to good health in advanced adulthood [1]. In these vulnerable age groups, behavioural patterns are learned, tested and often habituated. Lifestyles develop that continue into old age and become increasingly resistant to change [2]. Thus, childhood and adolescence are of particular importance for intervening measures promoting healthy growing up and detecting and treating diseases as early as possible. Accordingly, this applies not only to children and adolescents (C&A) without health restrictions, but also to C&A who already developed medical conditions. The timely use of prevention and early detection as well as the strengthening of paediatric care can be seen as important elements of quality-oriented care.

### Screening examinations

Classical (secondary) preventive approaches for C&A in Germany are particularly the so called “U examinations” for the early detection of health disorders and abnormalities in development, introduced in the 1970s. Up to the age of 6, the U1 to U9 are carried out, which consequently contribute to the necessary treatment and support of the families. In addition, C&A between the ages of 12 and 14 are again entitled to a preventive examination (J1). Additional examinations, such as the U10, U11 and J2, have long been recommended by paediatricians, but have not yet been included in the benefits catalogue of the statutory health insurance (Gesetzliche Krankenversicherung, GKV) [3]. In addition to the established examination, these early screening measures also include questionnaire concepts and medical consultation concepts, as demanded by the Professional Association of Paediatricians and Adolescent Physicians (Berufsverband der Kinder- und Jugendärzte, BVKJ) for all early screening measures [4]. While the participation rate in the early screening measures in childhood (U1-U9) is more than 90% in each case, this proportion decreases steadily in adolescence [5, 6]. Only 46–48% of young people make use of J1. There are clear regional differences, with the federal states of Berlin, Brandenburg and Mecklenburg-Western Pomerania lying in the federal average with 37–55% [7]. This typical age dependency is also evident in the use of paediatricians: The older the C&A, the more frequently families switch from paediatricians to general practitioners. On the one hand, this can be attributed to the fact that starting from age 11 self-initiated physician attendance increases and thus the decision of whether to attend a physician is no longer reserved to the respective parents alone. However, a predominantly self-determined access to outpatient care can be assumed at the earliest from the age of 16 [5]. On the other hand, the interruption between the statutorily determined last U9 in childhood (between 60th and 64th month of life) and the later J1 in adolescence (between 12 and 14 years) means that the latter is much less accepted. In particular, children between the ages of 11 and 13 have the lowest demands on outpatient medical care [5].

Eye diseases are rare in childhood, however if they occur they usually affect both eyes and can lead to severe visual impairment if not treated immediately. In the first years of life, ametropia, strabismus and amblyopia are among the most common visual diseases. The prevalence rates are 10, 5 and 3%, respectively, although these vary greatly depending on the cohort studied and the corresponding disease definition. This translates to 70,000, 35,000 and 21,000 new cases of C&A every year [8]. In addition, only 2–4% of C&A make use of ophthalmologists [9]. A review found that screening and therapy reduce the prevalence of common eye diseases in children [8]. The current U-examinations are not sufficient in standard care.

### Obesity

Over the past decades, overweight and severe overweight (obesity) among C&A have increased significantly. Nationwide, 15% of children younger than 17 are overweight, of which 6% are obese [6]. The main causes are wrong or unhealthy diets with a simultaneous lack of physical activity. For example, only 72% of children achieve the activity level of at least 60 min per day recommended by the World Health Organization [6]. Less than one-third of 11 to 15-year-old adolescents are regularly physically active [10]. At the same time, the consumption of sweets and beverages containing sugar is increasing [6]. Also genetic predispositions and early childhood socialisation in the family play a role. Only in the rare cases a primary disease is present [11]. The consequences of overweight/obesity are on the one hand subsequent vascular diseases, high blood pressure and diabetes, which contribute to the restriction of physical functions, and on the other hand the negative eating habits that usually translate into adulthood [11]. The economic costs associated with obesity in Germany are estimated at 10 to 15 billion € per year [12–14]. Against this background, a corresponding preventive measure is necessary.

### Intervention

In 2007, AOK Nordost, a regional health insurance in the federal states of Berlin, Brandenburg and Mecklenburg-Western Pomerania, developed a programme for the early detection of diseases in C&A. The programme“AOK-Junior” is organised on the basis of a selective contract, which means that it is an individual contract concluded between AOK Nordost and a single service provider, in this case the Professional Association of Paediatricians and Adolescent Physicians (Berufsverband der Kinder- und Jugendärzte [BVKJ]). Selective contracts contain additional medical services with individual health insurance providers that transcend the legally defined medical care. This contributes to the assessment of innovative treatment approaches and can serve as a test for the possible inclusion of a medical service in the in the statutory health care system [15]. The existing selective contract is based on the special care regulations. They are intended to expand the scope for the health insurance to design their own plans and to remove bureaucratic barriers to selective contracts. Contracts for innovative and effictive services will be made possible which have not yet found their way into the standard health care system. The special care contracts are intended to network different performance sectors with each other and to enable interdisciplinary, multidisciplinary care.

By taking part, AOK-Junior offers families the opportunity to identify and treat their children with diseases at an early stage in order to avoid secondary diseases in the long term. On the other hand, competencies can also be strengthened in order to develop health awareness as a prerequisite for a healthy life [6]. This is especially true if children are included in the programme from the very beginning. Pediatricians thus act as gatekeepers and can contribute to the improvement of comprehensive and holistic care for C&A. The funding agencies also have the opportunity to specifically adapt to the needs and problems of their insured population by means of a tailor-made selective contract [15].

The selective contract “Pediatric-centred integrated care AOK-Junior (AOK-Junior)” completes the legally defined medical care for insured persons up to the age of 18 in cooperation with qualified paediatricians. AOK-Junior focuses on health protection, early detection and prevention with the aim of maintaining C&A’ health and detecting and preventing diseases at an early stage. By participating in AOK-Junior, quality, service and coordination of treatment and prevention for C&A should be improved compared to standard care.

The AOK Nordost is responsible for preventive measures in addition to program development. The target group of this contract is therefore the entire population of children and young people insured with AOK Nordost (approx. 250,000) and their parents. The number of participating insured persons is approximately 65,000 C&A (August 2019). Thus, more than one fifth of the C&A insured under the AOK Northeast insurance participate in AOK-Junior.

Responsible for the implementation of the defined services is the BVKJ-Service GmbH. BVKJ-Service GmbH represents the interests of approx. 560 paediatricians (August 2019) participating in AOK-Junior.

### Medical services of the selective contract

At the centre of the health care provision for C&A within the framework of AOK-Junior is the paediatrician, who coordinates the general medical-paediatric and specialist care. AOK-Junior comprises the following modules:

- The performance module includes additional early detection examinations: The focus of the **U10** (age 7–8) is, among other things, the recognition of motor development disorders as well as developmental and behavioural disorders. The **U11** (age 9–10) includes, among other things, the recognition of school performance disorders, socialisation disorders, behaviour harmful to health as well as tooth, mouth and jaw anomalies. The focus of **J2** (age 16–17) is the recognition/treatment of puberty and sexuality disorders, posture disorders, goitre formation as well as diabetes prevention and counselling in questions of behaviour, socialisation, family and sexuality.
- Performance module **Allergic Rhinitis**: The module promotes specific immunotherapy (SIT) for AOK-Junior participants with allergic rhinitis. By using a reminder system, the high current dropout rate is intended to be reduced and the rate of successfully completed hyposensitizations increased.
- Performance module **Ophthalmologic Early Screening Examination**. The module is intended to promote the early detection of eye diseases, visual defects and strabismus in children between the ages of 32 and 42 months. An individual consultation should avoid eye damaging influences and behaviours.
- **Lung Check** performance module. The aim of the Lung Check module is to prevent a chronic respiratory disease, to provide information about possible therapies and to positively influence the course of the disease. The preventive care benefit can be claimed by 6- to 7-year-olds under certain conditions and includes an outpatient pneumological examination as well as a health education consultation.
- **Skin Check** performance module. The skin check includes additional measures for the early detection and treatment of chronic skin diseases. Individual consultations are intended to help avoid skin-damaging influences and behaviours. The first skin check is aimed at 2 to 5-year-olds, the second at 13 to 17-year-olds.
- Performance module **Dental Health**. AOK-Junior participants receive a subsidy if the premolars have to be sealed. In addition, professional dental cleaning for AOK-Junior participants with ongoing orthodontic treatment can be subsidized.
- Performance module **Target Agreement on Overweight**. In order to reduce the morbidity and mortality risk of overweight or obese AOK-Junior participants in adulthood, a target agreement for weight reduction is concluded with either the participants or one parent. It is also essential to provide information on overweight and obesity, their serious health consequences and weight reduction options. If the goals are achieved, there is a bonus gift for the AOK-Junior participants. The measure is intended to bring about a change in dietary and movement habits.

### Aim of the study

The evaluation project should provide information on the quality of AOK-Junior in order to derive recommendations for further developing of the care model. This is done on the levels of structure, process and outcome quality. Within the framework of a health economic evaluation, the cost-effectiveness of the service modules of the selective contract will also be evaluated. Overall, this is a complex and interdisciplinary intervention.

### Objectives of the study

The main question is intended to answer the question to what extent the AOK-Junior programme can detect diseases in C&A earlier. The global hypothesis tests whether the intervention (participation in the selective contract AOK-Junior) improves the effectiveness of early disease detection compared to standard care. Furthermore, it is assumed that the selective contract is superior in further effectiveness parameters (response, satisfaction) as well as in cost-effectiveness (costs, cost-effectiveness ratio).

The questions for the individual levels of the evaluation project are as follows:

- Structural quality: Which structures are used to implement AOK-Junior?
- Process quality: Which promoting and hindering factors can be identified for the implementation of the selective contract?
- Outcome quality: What effects does AOK-Junior have on the participants?
- Health economic evaluation: How high are the programme costs of AOK-Junior? What is the cost-effectiveness of the selective contract and individual service modules?
- Question for further development: Which recommendations for action can be derived for the further development of selective contractual care?

Furthermore the following hypotheses will be examined:

- H_1_: Diseases are discovered earlier in the intervention group (IG) than in the control group (CG).
- H_2_: More diseases are discovered in IG than in CG.
- H_3_: Early detection of disease in IG leads to earlier therapy than in CG.
- H_4_: In the IG, follow-up costs compared to the CG are avoided.
- H_5_: Early detection of disease is more cost-effective in the IG (cost per discovered case) than in the CG.

## Methods

### Study design

**Fig. 1:**
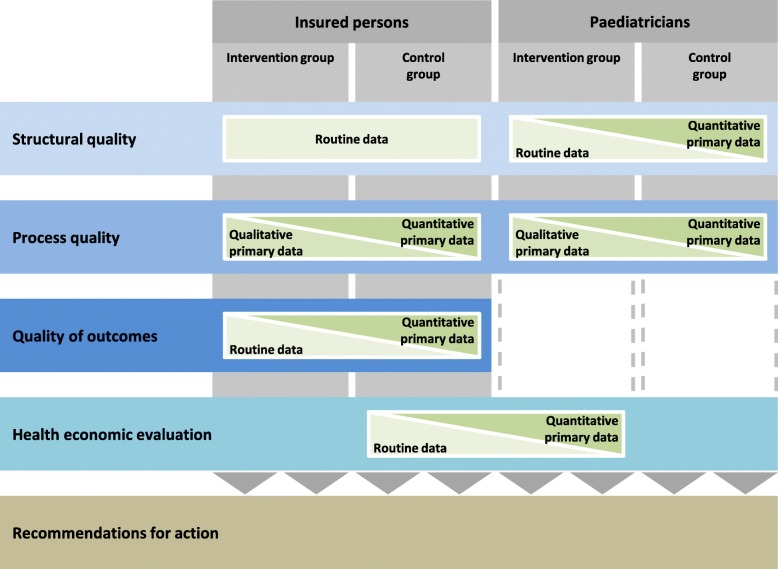
Study design

Since different research methods are necessary to answer the questions, the study design is differentiated according to the individual aspects of the evaluation (Fig. 1).

#### Evaluation of the structural quality

The evaluation of the structural quality is carried out on the basis of cross-sectional analyses of the available routine data. Reference data are used to evaluate the degree of achievement. In addition, primary data on structural features are collected as part of the quantitative cross-sectional survey of paediatricians. In this context, the cross-sectional design is well suited to examining the absolute and relative frequency of structural features between participating and non-participating physicians or insured persons. The structural characteristics of the physicians include, for example, region, group of specialists, size of practice, practice kind (single practice, joint practice or practice communities), number of patients treated per quarter, age and sex. Among the structural characteristics of insured persons are e.g. age, sex, nationality, income, education and occupational status (of parents) and chronic diseases (diabetes mellitus, ADHD, asthma, allergy and obesity).

#### Process evaluation

AOK-Junior performs a process analysis to evaluate the process quality. Based on this, a qualitative cross-sectional survey of doctors and insured persons is carried out using focus groups to determine satisfaction with the programme as well as potentials and barriers. The results will be verified by a cross-sectional survey using standardised questionnaires. This enables doctors and insured persons to be asked about the quality of the procedures, the scope of services, satisfaction with the programme, motivation and reasons for or against participation.

#### Evaluation of outcomet quality

The outcome quality is assessed from the routine data as a longitudinal study in the design of a cohort study. Cohort studies are well suited to examine the frequency and timing of a disease. As the AOK-Junior programme has been implemented since 2007 and routine data are available, data can be analysed over a longer period of time than is usual for prospective studies. In addition, practicable aspects speak in favour of the method: Overall costs are lower and the data are available more quickly. The outcome quality is assessed on the basis of the time of detection (see hypothesis 1) and the incidence rate (see hypothesis 2) of a chronic disease. The secondary preventive measure AOK-Junior aims at the early detection of disease. The control group is generated by propensity score matching (PS matching).

Further relevant result parameters are collected by means of quantitative cross-sectional surveys. For example, the health-related quality of life of C&A, which is assessed via KIDSCREEN-27 [16, 17]. As a result of the increase in chronic degenerative diseases, the health-related quality of life is becoming increasingly important as a outcome parameter [1, 2].

#### Health economic analysis

First, a cost study is carried out from the statutory health insurance perspective. The intervention costs include all costs associated directly with the selective contract. The results of the cost study and the quality of the results are then incorporated into a cost-effectiveness study. The cost-effectiveness-analysis is based on the costs per detected case. The analysis is carried out in three steps: (1) Calculation of the costs per detected case of all target diseases, whereby the total costs up to the identification and confirmation of the diagnosis are taken into account and (2) Calculation of the costs per detected case differentiated according to target disease. These analyses are carried out over the entire area of the AOK Nordost, while (3) a long-term analysis is limited to the federal state of Brandenburg (with the longest programme duration of 10 years). The aim is to analyse whether and how many months or years an AOK-Junior disease is diagnosed earlier and which follow-up costs can be avoided.

### Measurements

Within the scope of the project, the instruments for primary surveys shown in Table 1 with the parameters mentioned will be developed for the target groups. In addition, the performance and accounting data of AOK Nordost will be included in the analyses.

**Table 1:** Survey instruments and parameters

Target group	Instrument	Parameter
Paediatricians	Structured interview guide for the focus group survey	Barriers / hindering factors, potentials / promoting factors, satisfaction
	Partially standardised questionnaire	Participation, abortion reasons, practice size/form, specialist group, age (year of birth), gender, work experience, region, working time per week, number of patients treated per quarter, proportion of private patients, satisfaction, incentives, barriers / obstructive factors, potentials / promoting factors
Children and adolescents	Structured interview guide for the focus group survey	Barriers / hindering factors, potentials / promoting factors, satisfaction, expected benefit, effort
	Partially standardised questionnaire	Age (year of birth), gender, sociodemography (income, education, migration, occupational status), region, satisfaction, barriers / hindering factors, potentials / promoting factors, expected benefit / incentives, effort, utilization of U-examinations (U8, U9), health-related quality of life (KIDSCREEN 27), health behavior, body size and weight

### Ethical and scientific standards

In general, ethical and scientific standards in their current version are taken into account in the conduct of studies, above all the Memorandum on Safeguarding Good Scientific Practice of the German Research Foundation. For the analysis of the secondary data, “Good Practice Secondary Data Analysis” is used in particular. The primary data collection is based on the STROBE Statement [18]. For health economic evaluation, the methods of health economic evaluation in health care research and the Hanover Consensus [19] apply.

The quality assurance of the project is based on the guidelines for ensuring good epidemiological practice [20]. Prior to the evaluation, a detailed study plan with time and organisational procedures will be drawn up. The questionnaires designed for the study will be pre-tested and all data will be validated before evaluation.

### Study population

The underlying study population consists primarily of an IG and a CG at paediatricians as well as C&A level. The AOK-Junior selective contract is implemented in entire catchment area of the AOK Nordost. At the level of paediatricians, all participating physicians are included in the quantitative survey. The CG includes doctors who are also organised in the BVKJ but do not participate in the selective contract AOK-Junior. For the inclusion of the CG, it is considered that these are active in catchment areas that are as comparable as possible. A sub-sample will be taken for the qualitative surveys. Thereby, an adequate representation of the range of catchment areas of established paediatricians is taken into consideration.

At the level of the insured, the study population is drawn on the basis of routine data. The participants of the IG are C&A from birth up to and including the age of 17. Thus for each module its own population is needed. The exception is the target agreement on overweight. In the CG are C&A who are also insured with the AOK, but who do not participate in the AOK-Junior prevention programme. In order to ensure comparability between participating and non-participating insured persons, a CG is formed for the IG on the basis of routine data using the characteristics of sex, age and comparable region by means of PS matching.

### Participant recruitment

A random sample of all participating and non-participating physicians will be drawn and invited for the focus group survey of paediatricians. For the quantitative survey, all paediatricians from the three federal states of Berlin, Brandenburg and Mecklenburg-Western Pomerania will be invited to participate. The difficulty with this target group is in particular a low response rate. On the one hand, this was taken into account by a conservative assumption of the response rate (30%) in the case number calculation. On the other hand, in order to achieve a high acceptance of the target group, a joint address with the BVKJ was made. In addition, each questionnaire contains a personal address in the form of a handwritten note, which demonstrably leads to an increase in the response rate [4]. A reminder is sent to all physicians after 2 weeks and after 4 weeks. In addition, 5 tablets will be raffled as incentives. If no focus groups can be implemented due to insufficient willingness to participate, qualitative telephone interviews will be used as an alternative.

For the focus group survey of the C&A, a random sample of all participants and non-participants is selected and invited from the routine data of the AOK Nordost. For the quantitative survey, a random sample of the participating and non-participating C&A is also selected from the routine data of the AOK Nordost to receive a postal questionnaire. A higher response rate can be expected among C&A than with the target group of physicians. Nevertheless, it is difficult to convince the target group to participate in the surveys. On the one hand, this was taken into account by a conservative assumption of the response rate (40%) in the case number calculation. On the other hand, in order to achieve a high level of acceptance among the target group, a joint approach was made with the BVKJ. In addition, each questionnaire contains a personal address in the form of a handwritten note, with thanks for participating [4]. Proven methods such as incentives and reminders are used to increase response rates and reduce the risk of selection bias through systematic non-participation. In addition, a target group-specific and culture-sensitive approach and linguistic translation of the questionnaire instrument for Turkish, Russian and Arab migration groups will be provided. After 2 weeks and after 4 weeks a reminder will be sent to all C&A. In addition, 30 age-appropriate incentives of max. 30 € each will be raffled off.

### Sample size calculation

#### Qualitative surveys

The number of cases of the qualitative surveys depends on the method of theoretical sampling. The population is not selected according to representativity, but according to the theoretical significance for the research question. Thus, the sample size cannot be determined in advance. The inclusion of further cases is terminated when “theoretical saturation” [21] is reached. For focus groups a sample of 6–8 participants is recommended. Furthermore, following the recommendations 3 focus groups are expected for each target group (*n* = 4 groups with participating and non-participating physicians and insured persons) [22, 23]. This corresponds to a case number of *n* = 72–96 subjects.

#### Quantitative surveys

**Fig. 2:**
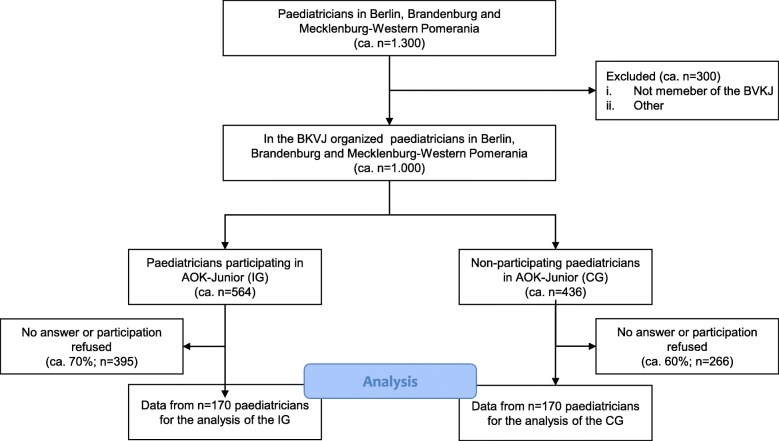
Flowchart for the primary survey among the target group of paediatricians based on the STROBE Statement

At the level of paediatricians, all physicians participating in AOK-Junior are included (*n* = 564). With a conservatively expected response rate of 30%, a net sample of *n* = 170 doctors is estimated. Furthermore, a control group of the same size is intended. Approximately 1000 paediatricians will be contacted to reach the sample. The expected course of the study for the target group is shown in Fig. 2.

**Fig. 3:**
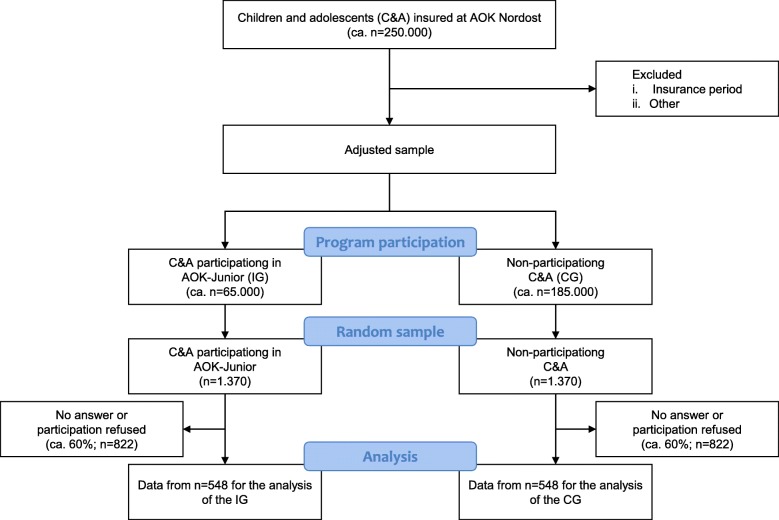
Flowchart for the primary survey of the children and adolescents target group based on the STROBE statement

At the level of the C&A, all patients who have used the services are included in the IG as part of the secondary data analyses. Using PS matching, a suitable CG is drawn from the secondary data in a ratio of 1:1. The collection of primary data takes place in a sub-sample, whose case number was calculated on the basis of the primary outcome parameter of the health-related quality of life. A 2 × 3-factorial design is used to compare the intervention with standard care (IG/CG) (factor 1), taking into account multiple factors, e.g. the region (factor 2). To demonstrate expected small effects at the significance level of α ≤ 0.05 and a power of 80%, a net sample of *n* = 1096 (*n* = 548 per group) is required for analysis. With a conservatively expected response rate of 40%, a sample of *n* = 2740 is invited to participate. The case number planning and the analysis methods are selected in order to adjust for covariates (e.g. gender). The expected course of the study for the target group is shown in Fig. 3.

The participation in the scientific investigation and the information are voluntary for the paediatricians as well as the C&A and their parents. The primary data is collected by the evaluator’s staff. The inclusion of the subjects is carried out after complete clarification and presentation of a written declaration of consent. Participants can revoke their consent at any time. The evaluation of the data is pseudonymised.

The primary data collected and the secondary data used are stored in pseudonymised form on a secure, encrypted network drive at the Hannover Medical School (MHH) and the access rights are limited to the project staff. A transmission or passing on of data to third parties is excluded. No information is transmitted to third parties that could allow conclusions to be drawn about individual persons. All data will be stored on a secure, encrypted hard disk after completion of the study and deleted after 10 years at the earliest (in accordance with the recommendations on safeguarding good scientific practice).

### Statistical analysis

#### Qualitative analyses

The qualitative interviews are recorded with dictation machines and then transcribed. The analysis of the transcripts follows the procedure of inductive category development as well as the qualitative, summarizing content analysis according to Mayring [24]. The data is evaluated with the MAXQDA software.

#### Quantitative analyses

The data evaluations are initially performed descriptively. The hypotheses developed are also tested for inferential statistics. In order to ensure comparability between participating and non-participating insured persons, a CG is formed for the IG on the basis of the routine data using the characteristics gender, age and comparable region. Participants from both groups are brought together with PS matching at the individual level. The analyses of the primary data take into account in particular covariates for which it was not possible to match at the secondary data level, such as socio-economic status and migration background. The influence of the selective contract is checked using ANCOVA. The analyses are carried out with the software programs SAS, SPSS and Microsoft Excel.

#### Health economic analyses

Within the scope of a complete health economic evaluation, the incremental costs are related to the incremental effects. In addition, determinants for achieving an appropriate cost-effectiveness ratio are examined.

## Discussion

The project provides information on the extent to which secondary prevention measures can lead to early detection of diseases and a reduction in unhealthy behaviour. Furthermore, the evaluation shows potentials and barriers during the implementation of the selective contract. For the first time, a cost analysis enables more concrete assessments of the implementation of future prevention measures, the reduction of childhood and youth specific diseases and creates a basis for future decisions and investments in the areas of health promotion and prevention. In addition, determinants for achieving an appropriate cost-effectiveness ratio are investigated. The evaluation results are expected to provide indications for the further development of the selective contract,for which recommendations for action will be derived.

The aim is to make the results and concrete recommendations available to the implementing actors. Thus, the gap in health care provision for C&A should be closed and the quality of prevention, early detection and prevention improved. The evaluation project can contribute to reducing the misuse, underuse or overuse of health care services and to meeting the needs and demands of C&A. An extension of selective contractual services or care approaches to standard care can also increase patient satisfaction.

## Data Availability

Not applicable.

## Abbreviations

AOK: Allgemeine Ortskrankenkasse (Health Insurance Fund)
BVKJ: Berufsverband der Kinder- und Jugendärzte (Professional Association of Paediatricians and Adolescent Physicians)
C&A: Children and adolescents
CG: Control group
IG: Intervention group
